# 564 Clearing the Air: The Impact of COPD on Inhalation Injury Outcomes

**DOI:** 10.1093/jbcr/iraf019.193

**Published:** 2025-04-01

**Authors:** Christopher Fedor, José Arellano, Mare Kaulakis, Hilary Liu, Alain Corcos, Garth Elias, Matthew Siedsma, Jenny Ziembicki, Francesco Egro

**Affiliations:** University of Pittsburgh School of Medicine; University of Pittsburgh Medical Center; University of Pittsburgh School of Medicine; University of Pittsburgh Medical Center; University of Pittsburgh Medical Center; University of Pittsburgh Medical Center; University of Pittsburgh Medical Center; University of Pittsburgh Medical Center; University of Pittsburgh Medical Center

## Abstract

**Introduction:**

Chronic obstructive pulmonary disease (COPD) is characterized by obstruction of small airways (chronic bronchitis) and emphysema, which lead to air trapping and shortness of breath in response to physical exertion. Such a disease process can inhibit the vital physiologic functions that are necessary to keep the lungs healthy and free of pathogens. In patients with inhalation injuries, where there can be significant airway damage, COPD patients may thus be less equipped to heal. This study therefore examined the effect of COPD comorbidity on clinical outcomes for patients with inhalation injuries.

**Methods:**

We conducted a retrospective analysis of patient records from a single tertiary care ABA-certified burn center (January 2012-January 2024). Fiberoptic bronchoscopy was used to confirm diagnosis of inhalation injury, and flash burns caused from smoking on home oxygen therapy were excluded. Pulmonary function test (PFT) data were extracted to rank COPD severity using the Global Initiative for Obstructive Lung Disease (GOLD) criteria. Major outcome variables included percentage of total body surface area with second- or third-degree burns (TBSA), carboxyhemoglobin (COHb) at presentation, complications, and hospital length of stay.

**Results:**

Of 184 patients with confirmed inhalation injury, 69 (37.1%) patients had COPD. COPD patients were older (60 ± 14 years, p< 0.001) and more likely to be a current smoker at the time of injury (73%, p< 0.001). External injury, measured by TBSA, was similar in both groups (p=0.244). Intubation timing (p=0.926), COHb at presentation (p=0.248), hospital days (p=0.631), and complication rates (p=0.646) were also similar between groups. There was no difference in mortality rate (p=0.748). However, in a subgroup analysis of patients who died, COPD patients survived an average of 7.3 days longer post-injury compared to non-COPD patients, after adjusting for age, COHb, and TBSA (SE=3.17, 95%CI [0.82, 13.8], p=0.029).

**Conclusions:**

COPD did not worsen hospital outcomes in inhalation injury patients. Injury severity was a stronger correlate, suggesting that the magnitude and acuteness of these injuries may overshadow the added risks posed by COPD in the critical care setting. Moreover, COPD patients who died experienced slower functional decline, possibly due to protective effects of this population’s prescription respiratory therapies or improved handling of respiratory stress given baseline lung compromise. Overall, COPD had no significant effect on the immediate clinical outcomes following smoke inhalation injuries.

**Applicability of Research to Practice:**

Smoke inhalation injuries remain a significant challenge in burn care, with no universally effective treatments available to date. Working towards understanding who is particularly at risk—especially when chronic illnesses are concerned—will be important for standardizing protocols and prognostication.

**Funding for the Study:**

N/A

